# The ability of apolipoprotein E fragments to promote intraneuronal accumulation of amyloid beta peptide 42 is both isoform and size-specific

**DOI:** 10.1038/srep30654

**Published:** 2016-08-01

**Authors:** Ioannis Dafnis, Letta Argyri, Marina Sagnou, Athina Tzinia, Effie C. Tsilibary, Efstratios Stratikos, Angeliki Chroni

**Affiliations:** 1 Institute of Biosciences and Applications, National Center for Scientific Research “Demokritos”, Agia Paraskevi, Athens 15310, Greece; 2 Protein Chemistry Laboratory, Institute of Nuclear and Radiological Sciences and Technology, Energy and Safety, National Center for Scientific Research “Demokritos”, Agia Paraskevi, Athens 15310, Greece

## Abstract

The apolipoprotein (apo) E4 isoform is the strongest risk factor for late-onset Alzheimer’s disease (AD). ApoE4 is more susceptible to proteolysis than apoE2 and apoE3 isoforms and carboxyl-terminal truncated apoE4 forms have been found in AD patients’ brain. We have previously shown that a specific apoE4 fragment, apoE4-165, promotes amyloid-peptide beta 42 (Aβ42) accumulation in human neuroblastoma SK-N-SH cells and increased intracellular reactive oxygen species formation, two events considered to occur early in AD pathogenesis. Here, we show that these effects are allele-dependent and absolutely require the apoE4 background. Furthermore, the exact length of the fragment is critical since longer or shorter length carboxyl-terminal truncated apoE4 forms do not elicit the same effects. Structural and thermodynamic analyses showed that apoE4-165 has a compact structure, in contrast to other carboxyl-terminal truncated apoE4 forms that are instead destabilized. Compared however to other allelic backgrounds, apoE4-165 is structurally distinct and less thermodynamically stable suggesting that the combination of a well-folded structure with structural plasticity is a unique characteristic of this fragment. Overall, our findings suggest that the ability of apoE fragments to promote Aβ42 intraneuronal accumulation is specific for both the apoE4 isoform and the particular structural and thermodynamic properties of the fragment.

Amyloid peptide beta (Aβ) has been proposed to play a key role in the pathogenesis of Alzheimer’s disease (AD). The Aβ peptides can vary in length; most of Aβ peptide produced contains 40 amino acids (Aβ40), whereas a small proportion contains 42 amino acids (Aβ42)^1^,^2^. Aβ42 is more hydrophobic, more prone to fibril formation and more closely associated with the pathogenesis of AD than shorter Aβ forms^1^,^2^. The amyloid hypothesis for AD postulates that Aβ is deposited extracellularly in AD patients’ brain, forms soluble oligomers that affect synaptic structure and plasticity and that long insoluble amyloid fibrils accumulated in senile plaques lead to widespread neuronal dysfunction and ultimate cell death^2^,^3^. However, numerous studies on postmortem AD and mild cognitively impaired patients and on transgenic mouse models have shown the presence of Aβ inside neurons, with Aβ42 being the majority of intraneuronal Aβ^4^. Furthermore, the intraneuronal Aβ accumulation was shown to precede the formation of senile plaques and implicated in the onset of early cognitive alterations contributing to the pathological cascade of events that lead to neuronal dysfunction and eventually to AD^4^,^5^,^6^,^7^.

The strongest and best-established genetic risk factor for sporadic late-onset AD is the apolipoprotein E (*APOE*) ε4 allele^8^,^9^. ApoE is a major protein of the human lipoprotein transport system in the circulation and brain^10^,^11^. The apoE gene is expressed highly in liver and the brain^12^. In brain, apoE is synthesized primarily by astrocytes and to a lesser extent by microglia and neurons^10^. ApoE contains 299 residues and has three common isoforms (apoE2, apoE3, apoE4) that differ in the amino acid positions 112 and 158^11^. ApoE3 has a cysteine at residue 112 and an arginine at residue 158, whereas apoE4 has an arginine and apoE2 has a cysteine at each position. It has been demonstrated that apoE4 is much more susceptible to proteolysis than apoE3 and apoE2 and carboxyl-terminal truncated forms of apoE4 have been found in brains of AD patients and apoE4 transgenic mice^13^,^14^,^15^,^16^,^17^,^18^. The apoE4 fragmentation has been proposed to be an early event in the pathogenesis of AD^13^. A previous study from our lab showed that a specific carboxyl-terminal truncated apoE4 fragment, apoE4[Δ(166–299)] (designated hereafter as apoE4-165) with a molecular weight of 19 kDa (a size within the range of molecular weights of carboxyl-terminal truncated apoE4 fragments found in brains of AD patients^14^,^15^,^19^) can promote the cellular uptake and accumulation of Aβ42 and leads to formation of reactive oxygen species (ROS)^20^. A longer length fragment, apoE4[Δ(186–299)] (designated as apoE4-185), or full-length apoE4 failed to elicit this effect, suggesting that not all apoE4 variants are equally bioactive^20^. Those findings provided an association between two molecular events, the proteolysis of apoE4 and the intraneuronal presence of Aβ, both of which are considered to be early events in the pathogenesis of AD.

ApoE is highly helical and has a labile tertiary structure that can assume structures that resemble a molten globule^21^. Lipid-free apoE is folded into two seemingly independent structural domains separated by a hinge region^22^,^23^. The amino-terminal region (residues 1–191) forms a four-helix bundle spanning residues 24–164 that segregates the hydrophobic core of the four helices from the solvent^24^,^25^. The carboxyl-terminal region (residues 216–299) is also highly α-helical, but its tertiary structure is very polymorphic and participates in inter-domain interactions with the amino-terminal domain as well as with lipids^26^. ApoE4 has been described to have both structural and functional differences to apoE2 and apoE3 and some of these differences have been attributed to different interactions between the amino-terminal and carboxyl-terminal domains of the molecule^26^,^27^. Several studies have suggested that the structural and biophysical properties of apoE can dictate the function of the protein and have provided insight into the mechanisms through which apoE is involved in cardiovascular and neurological diseases^26^,^27^,^28^,^29^,^30^.

Given our previous finding regarding the ability of apoE4-165 to promote intracellular accumulation of Aβ42, we asked whether the allelic state or fragment length are important factors for this function. Towards this goal, we analyzed the role of allelic background on the ability of apoE-165 fragments to promote Aβ42 internalization. Furthermore, we examined whether other longer or shorter-length fragments in the ε4 allelic background, namely apoE4[Δ(272–299)] (designated as apoE4-271), apoE4[Δ(147–299)] (designated as apoE4-146), apoE4[Δ(125–299)] (designated as apoE4-124), share similar functional properties with apoE4-165. The apoE4-271 fragment has been proposed to be present in the brain of AD patients, to induce neurofibrillary tangle-like intracellular inclusions in cultured neurons, to clear inefficiently Aβ peptides and to elicit AD-like neurodegeneration and behavioral deficits in transgenic mice^14^,^15^,^31^. Furthermore, it was proposed that apoE4-271 is cleaved further to generate 14–20 kDa fragments, displaying a fragmentation pattern similar to this observed in AD patients’ brain^14^. The apoE4-124 and apoE4-146 fragments have molecular weights (15 and 17 kDa, respectively) that also fall within the range of molecular weights of carboxyl-terminal truncated apoE4 fragments reported to be present in AD patients’ brains (14–22 kDa)^14^,^15^. Overall, our analysis demonstrates that the ability of apoE4-165 to promote the uptake and accumulation of Aβ42 in human neuroblastoma SK-N-SH and primary mouse neuronal cells is unique to this combination of fragment length and ε4 allelic background. None of the other fragments tested shared this property indicating that both length and allelic background are crucial for this function. Analysis of structural and thermodynamic properties of the truncated apoE variants suggested that apoE4-165 presents a unique combination of structural integrity and conformational plasticity that may underlie its unique functional properties.

## Results

### Protein expression and purification

**Figure 1:**
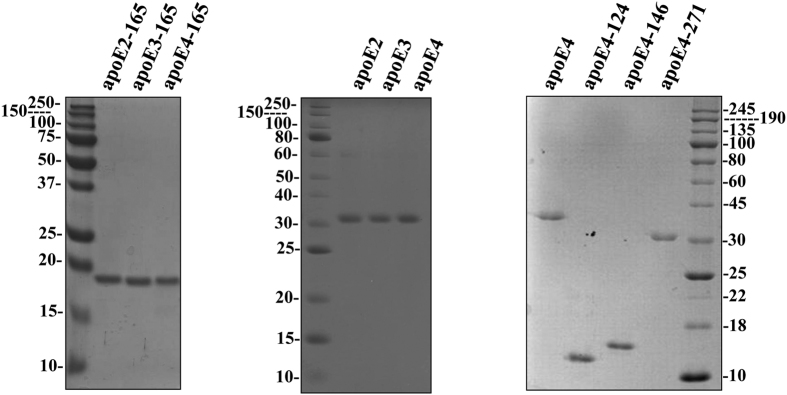
SDS-PAGE analysis of purified full-length and truncated apoE forms. The refolded full-length and truncated apoE forms, indicated on top of each panel, produced and purified as described under “Methods”, were subjected to electrophoresis on 15% SDS polyacrylamide gels and stained with Coomassie Brilliant Blue.

To investigate whether the allelic background of apoE-165 fragment affects the functional and structural properties of the protein, we expressed and purified the recombinant apoE2-165, apoE3-165 and apoE4-165 truncated forms, as well as all three apoE isoforms using an *E. coli* expression system established previously^32^,^33^. To address whether fragment length is crucial for apoE function, we produced, using the same expression system, the recombinant apoE4 truncated forms apoE4-271, apoE4-146 and apoE4-124. All proteins were expressed in soluble form and purified by nickel-nitrilotriacetic acid (Ni-NTA) chromatography as a fusion protein with thioredoxin. The fusion construct was subsequently cleaved by 3C protease, and the released apoE was isolated using a second Ni-NTA purification step. All proteins were at least 95–98% pure, as judged by SDS-PAGE analysis (Fig. 1).

### Effect of allelic background of apoE-165 fragments on the cellular uptake of Aβ42 by SK-N-SH and primary mouse neurons

**Figure 2:**
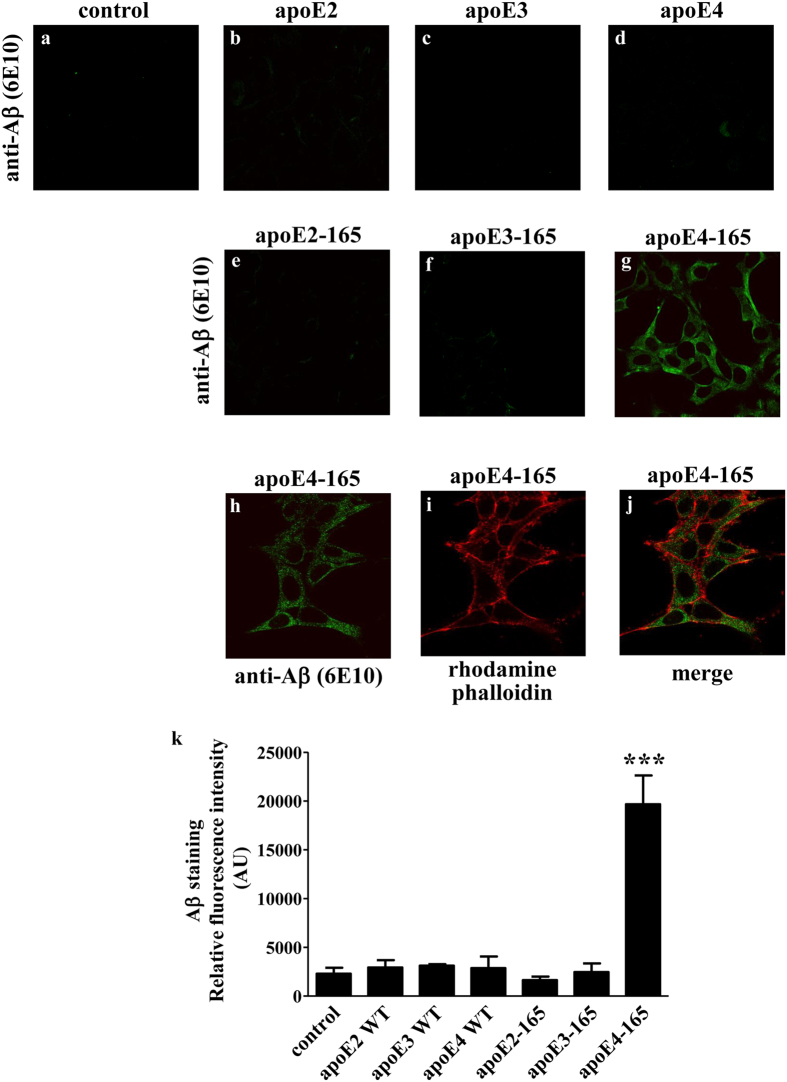
Fluorescence confocal laser scanning microscopy of SK-N-SH cells incubated in the presence of Aβ42 and full-length apoE or truncated apoE-165 forms. SK-N-SH cells were incubated with 25 ng/ml Aβ42 in the absence (control) or presence of 375 nM lipid-free full-length apoE or apoE-165 forms for 24 h, as indicated in each panel. Aβ immunostaining of cells was detected with the antibody 6E10 followed by a FITC-conjugated secondary antibody (a-h, green). F-actin was stained with rhodamine phalloidin (i, red). The merge of images h and i is shown in panel j. The quantitation of Aβ42 staining in cells based on relative fluorescence intensity measurements is shown in panel k. Values are the means ± SD (n = 3–7). ***p < 0.0001 vs control; AU: arbitrary units.

In a previous study we demonstrated that the apoE4-165 fragment can induce Aβ42 peptide uptake by SK-N-SH cells^20^. To address whether this property is allele-dependent we now examined whether apoE2-165 and apoE3-165 have the same effect on SK-N-SH cells. The apoE-165 forms were used at a concentration similar to the reported apoE concentration in human cerebrospinal fluid (365–396 nM)^34^,^35^. When SK-N-SH cells incubated with Aβ42 and lipid-free full-length apoE or truncated apoE-165 forms for 24 hrs, only apoE4-165 resulted in internalization of Aβ42, as indicated by the strong Aβ immunoreactivity of cells treated with apoE4-165 (Fig. 2g,h,k). Staining with the cytoskeletal F-actin marker rhodamine-phalloidin, that traces the outline of individual cells, indicated that Aβ42 resides primarily in the cytosol of the cells (Fig. 2h–j). No Aβ42 uptake was evident when full-length apoE isoforms, apoE2-165 or apoE3-165 were used or in the absence of any apoE forms, as indicated by the minimal Aβ immunoreactivity of cells (Fig. 2a–f,k).

**Figure 3:**
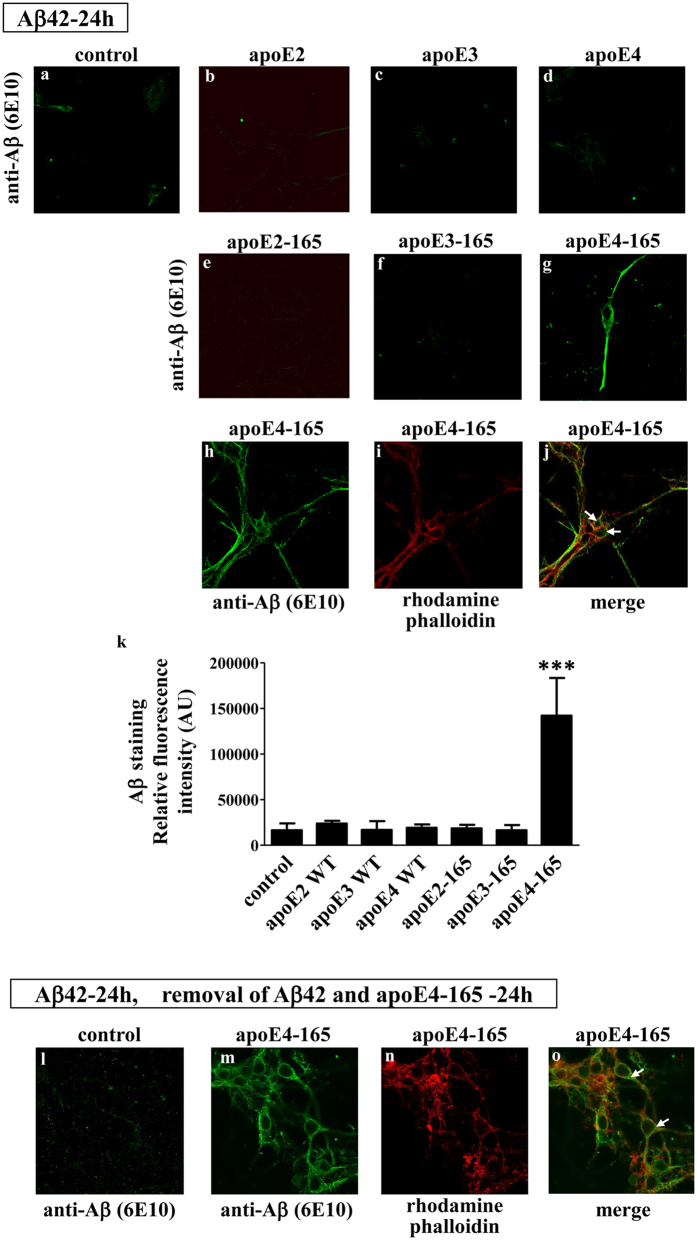
Fluorescence confocal laser scanning microscopy of primary mouse cortical neurons incubated in the presence of Aβ42 and full-length apoE or truncated apoE-165 forms. Primary mouse cortical neurons were incubated with 25 ng/ml Aβ42 in the absence (control) or presence of 375 nM lipid-free full-length apoE or apoE-165 forms for 24 h, as indicated in each panel (a–j). Primary mouse cortical neurons were also incubated with 25 ng/ml Aβ42 in the absence (control) or presence of 375 nM lipid-free apoE4-165 for 24 h and then washed and incubated further in fresh medium without Aβ42 or apoE4-165 for 24 more hours, as indicated (l-0). Aβ immunostaining of cells was detected with the antibody 6E10 followed by a FITC-conjugated secondary antibody (a-h, l, m, green). F-actin was stained with rhodamine phalloidin (i, n, red). The merge of images h, i and m, n is shown in panels j and o, respectively. The quantitation of Aβ42 staining in mouse cortical neurons, incubated with 25 ng/ml Aβ42 in the absence (control) or presence of 375 nM lipid-free full-length apoE or apoE-165 forms for 24 h, based on relative fluorescence intensity measurements is shown in panel k. Values are the means ± SD (n = 4–8). ***p < 0.0001 vs control; AU: arbitrary units. Two images for each experimental condition (i.e. incubation of primary mouse cortical neurons with 25 ng/ml Aβ42 in the absence (control) or presence of 375 nM lipid-free full-length apoE or apoE-165 forms for 24 h) showing the Aβ immunostaining of cells are presented in Supplemental Figure 1. In addition, F-actin staining of cells using rhodamine phalloidin or greyscale images of increased brightness/contrast are shown in Supplemental Figure 1, to facilitate the visualisation of cells outline, especially in the images with very low Aβ immunostaining.

To extend our findings to a more physiologically-relevant cellular system, we incubated primary mouse cortical neurons with Aβ42 and either full-length apoE or truncated apoE-165 for 24 hrs. Similar to the SK-N-SH cells, only apoE4-165, but not apoE2-165, apoE3-165 or full-length apoE induced the uptake of Aβ42 (Fig. 3a–hk). Furthermore, Aβ42 accumulation inside the cells persisted even 24 hrs post removal of both Aβ42 and apoE4-165 from the medium (Fig. 3l,m). Staining with rhodamine-phalloidin indicated some cytosolic localization of Aβ42, with the majority of the Aβ42 localizing in the neurites, as described previously for primary mouse cortical neurons incubated with Aβ42^36^ (Fig. 3h–j,m–o). These findings suggest that the ability of apoE4-165 fragment to promote the intraneuronal accumulation of Aβ42 is allele-dependent.

### Effect of allelic background of apoE-165 fragments on ROS formation by SK-N-SH in the presence of Aβ42

**Figure 4:**
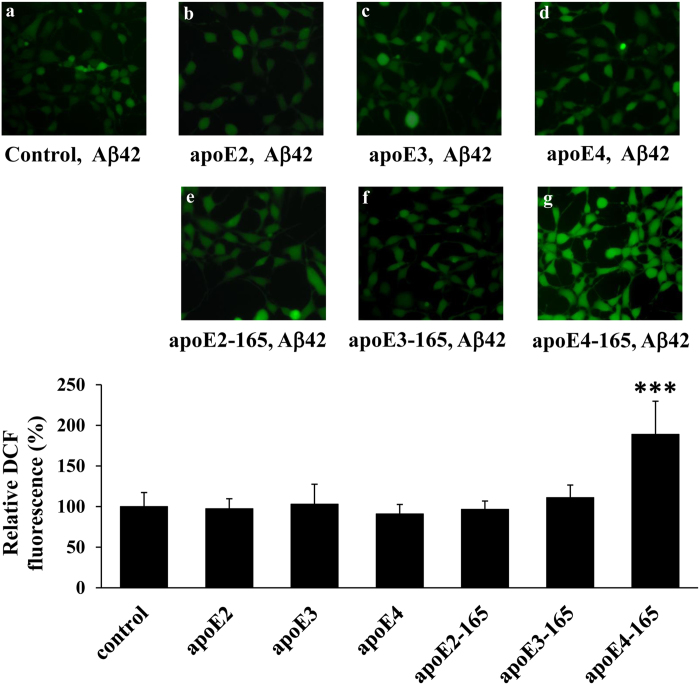
Effect full-length apoE or truncated apoE-165 forms in the presence of Aβ42 on ROS formation by SK-N-SH cells. SK-N-SH cells were incubated with 25 ng/ml Aβ42 in the absence (control) or presence of 375 nM lipid-free full-length apoE or apoE-165 forms for 24 h, as indicated in each panel. At the end of each incubation period the cells were incubated with DCFH-DA for 45 min. The formation of ROS was measured by detection of fluorescent DCF emitted from cells using a fluorescence microscope, as described under “Methods”. The DCF fluorescence of cells incubated with lipid free full-length apoE or apoE-165 forms and Aβ42 is shown relative to DCF fluorescence of control cells set as 100%. DCF fluorescent intensity was measured for at least 40 cells from the fluorescent images of each sample, as described under “Methods”, and the relative fluorescent intensity was taken as average of the values of at least 5 images for each experiment. Values are the means ± SD (n = 20) of four experiments. ***p < 0.0001 vs control.

We have previously shown that the apoE4-165-induced Aβ42 uptake by SK-N-SH cells leads to an increase of intracellular ROS, which are markers of oxidative stress^20^. To examine if this effect is also allele-dependent, we measured intracellular ROS formation using a specialized fluorescent probe as described under “Methods”. Incubation of SK-N-SH cells with Aβ42 and physiological concentrations of lipid-free full-length apoE or truncated apoE-165 forms for 24 hrs resulted in a significant increase of intracellular ROS only when the cells had been incubated with apoE4-165, whereas apoE2-165, apoE3-165 or full-length apoE isoforms had no such effect (Fig. 4). The finding that Aβ42 internalization and ROS formation are both specifically induced by apoE-165 in the same background of apoE4, further links these two phenomena and supports the hypothesis that Aβ42 internalization is the causative factor behind ROS formation.

### Effect of various carboxyl-terminal truncated apoE4 fragments on the cellular uptake of Aβ42 by SK-N-SH

**Figure 5:**
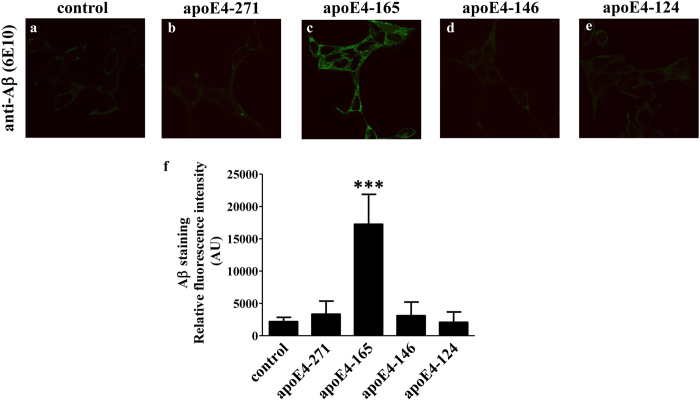
Fluorescence confocal laser scanning microscopy of SK-N-SH cells incubated in the presence of Aβ42 and carboxy-terminal truncated apoE4 forms. SK-N-SH cells were incubated with 25 ng/ml Aβ42 in the absence (control) or presence of 375 nM lipid-free carboxyl-terminal truncated apoE4 forms for 24 h, as indicated in each panel. Aβ immunostaining of cells was detected with the antibody 6E10 followed by an FITC-conjugated secondary antibody (green). The quantitation of Aβ42 staining in cells based on relative fluorescence intensity measurements is shown in panel f. Values are the means ± SD (n = 4-8). ***p < 0.0001 vs control; AU: arbitrary units. Two images for each experimental condition (i.e. incubation of SK-N-SH cells with 25 ng/ml Aβ42 in the absence (control) or presence of 375 nM lipid-free carboxyl-terminal truncated apoE4 forms for 24 h) showing the Aβ immunostaining of cells are presented in Supplemental Figure 2. In addition, F-actin staining of cells using rhodamine phalloidin is shown in Supplemental Figure 2, to facilitate the visualisation of cells outline, especially in the images with very low Aβ immunostaining.

Several studies to date have demonstrated the presence of variable-sized apoE4 fragments in brains of AD patients and apoE4 transgenic mice^13^,^14^,^15^,^16^,^17^,^18^. We have previously shown that a slightly larger-compared to the apoE4-165-fragment, namely apoE4-185, does not share the ability of apoE4-165 in promoting Aβ42 internalization^20^. We therefore asked whether, in addition to the allelic state, the specific size of apoE fragments is also important. To address this, we characterized three other apoE4 fragments of varying lengths that fall within the range of molecular weights of apoE4 fragments found in AD patients’ brains, namely apoE4-271, apoE4-146 and apoE4-124. These fragments sample a wide range of possible fragment lengths and allow us to investigate whether progressive carboxyl-terminal deletions generate gain-of-function apoE variants. Incubation of SK-N-SH cells with Aβ42 and physiological concentrations of lipid-free apoE4-271, apoE4-165, apoE4-146 or apoE4-124 fragments for 24 hrs showed that only apoE4-165 induced the Aβ42 cellular uptake (Fig. 5). These findings suggest that the length, in addition to the allelic background of apoE fragments, is a critical parameter to their ability to promote intraneuronal Aβ42 accumulation. It should be noted that amongst all fragments examined, apoE4-165 was found to be unique in the context of this property.

### Effect of allelic background on the structural and thermodynamic properties of apoE-165 fragments

**Figure 6:**
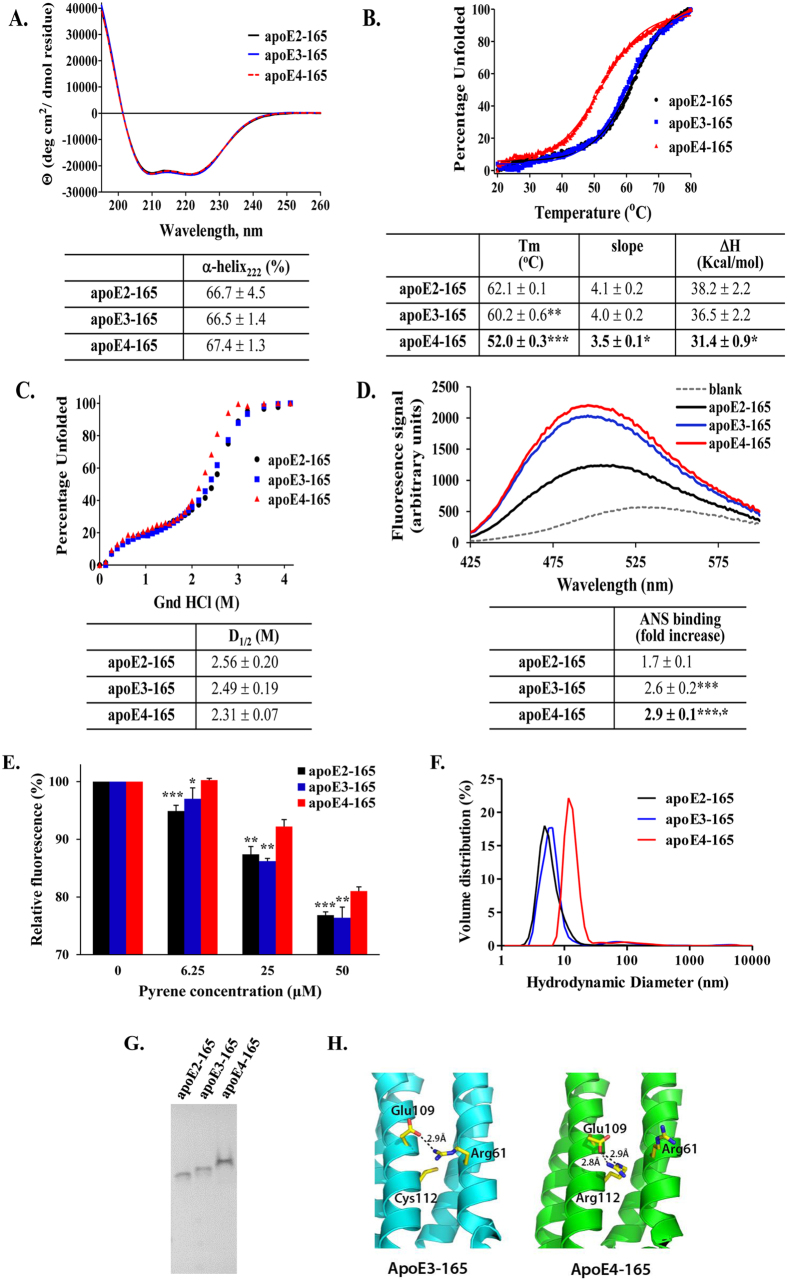
Physicochemical properties of truncated apoE-165 forms. (**A**) Far UV circular dichroism spectra of apoE-165 forms. Spectra are averages of three separate experiments. The % helical content was calculated based on the molar ellipticity at 222 nm as described under “Methods”. (**B**) Thermal denaturation profiles of apoE-165 forms. Y-axis has been normalized to correspond to the percentage of the protein in the unfolded state. Experimental data were fit to a simple two-state Boltzman transition (solid line). Apparent Tm and ΔH values were calculated as described under “Methods”. “Slope” is the calculated slope of the linear component of the thermal denaturation transition around Tm. Tm: **p < 0.005 apoE3-165 vs apoE2-165, ***p < 0.0001 apoE4-165 vs apoE2-165 and apoE3-165; slope: *p < 0.05 apoE4-165 vs apoE2-165 and apoE3-165; ΔH: *p < 0.05 apoE4-165 vs apoE2-165 and apoE3-165 (**C**) Chemical denaturation profiles of apoE-165 forms. Y-axis has been normalized to correspond to the percentage of the protein in the unfolded state. Apparent D_1/2_ values were calculated as described under “Methods”. (**D**) ANS fluorescence spectra in the presence or absence of apoE-165 forms. Spectra are the average of three separate measurements. Fold-increase is the increase in ANS fluorescence in the presence of the protein relative to free ANS in the same buffer. ***p < 0.0001 apoE4-165 and apoE3-165 vs apoE2-165, *p < 0.05 apoE4-165 vs apoE3-165 (**E**) Tryptophan fluorescence of apoE-165 forms in the presence of increasing concentrations of pyrene is shown relative to the fluorescence in the absence of pyrene (set as 100%). Values are the means ± SD of four experiments. *p < 0.05, **p < 0.005, ***p < 0.0001 for apoE2-165 or apoE3-165 vs apoE4-165 (**F**) Volume-normalized particle distribution profiles of apoE-165 forms, measured by DLS. (**G**) Native 15% PAGE analysis of apoE-165 forms. The gel was stained with Coomassie Brilliant Blue. Mobility differences between alleles are consistent with changes to overall protein charge due to the allelic background. (**Η**) Schematic representation of the differences in salt-bridge interactions between helices 2 and 3 in apoE3-165 and apoE4-165 based on the crystal structures of apoE3-165 (pdb code 1OR3) and apoE4-165 (pdb code 1GS9).

ApoE is a thermodynamically unstable protein that exhibits high conformational plasticity that is important for its function^21^,^26^. Changes in the structural and thermodynamic properties of apoE have been correlated with changes in its function in many studies^26^,^27^,^28^,^29^,^37^. To examine whether the structural and thermodynamic properties of apoE-165 are affected by its allelic state we compared the properties of apoE2-165, apoE3-165 and apoE4-165 in the context of secondary structure, thermodynamic stability and hydrophobic surface solvent exposure (Fig. 6A–D). All three fragments were found to be highly helical with no significant differences in secondary structure (Fig. 6A). Furthermore, all three fragments underwent a cooperative unfolding transition during thermal denaturation (Fig. 6B). The transition point of apoE4-165 however (indicated by the T_m_ parameter), was found to be much lower than the other two alleles, up to 10 °C. Accordingly, the calculated apparent ΔH value of apoE4-165 was up to 6.8 kcal/mol lower, indicating that compared to the other two alleles, apoE4-165 is thermodynamically destabilized. Similarly, apoE4-165 has a slightly less cooperative transition compared to the other two alleles, as evidenced from the slightly smaller value of the slope of the transition mid-point (3.5 versus 4.0 and 4.1, Fig. 6B). ApoE4-165 was also found to be slightly more sensitive versus chemical denaturation (Fig. 6C) and presented an increased amount of hydrophobic exposure to the solvent compared to the other two alleles (Fig. 6D).

To further examine any structural alterations in the apoE4-165 fragment we compared the pyrene-induced quenching of the tryptophan residues in apoE-165 for each allele (Fig. 6E). Both apoE2-165 and apoE3-165 tryptophan fluorescence was quenched to a similar degree whereas the reduction in fluorescence was significantly less for apoE4-165, indicating that at least one of the four tryptophan residues of this fragment has a different degree of exposure to the solvent. Measurement of the hydrodynamic radius of the three fragments by Dynamic Light Scattering (DLS) indicated that apoE4-165 has a higher hydrodynamic radius compared to the other two alleles (Fig. 6F) although it does not appear to be prone to aggregation as indicated by native-gel analysis (Fig. 6G). Taken together these observations suggest that the allelic content in apoE-165 can induce structural changes as well as thermodynamic changes.

In an effort to understand the unique properties of apoE4-165 in a structural context we examined existing crystal structures of this fragment. Visual inspection of available crystal structures of apoE3-165 and apoE4-165 indicates that the only amino acid difference between these two alleles (Arg112 for ε4 vs. Cys112 for ε3 and ε2) leads to the abrogation of an electrostatic salt-bridge interaction between Glu109 and Arg61 only in apoE4-165 that should destabilize the interaction between Helix 2 and Helix 3 of the protein (Fig. 6H). The same applies for the comparison between apoE4-165 and apoE2-165 since they also differ at that particular amino acid. Interestingly, this allele-dependent change has been proposed before to account for different inter-domain interactions between apoE3 and apoE4, primarily though the unavailability for Arg61 to interact with the carboxyl-terminal domain in apoE3. Our results suggest that the ε4 allele not only affects the interactions of the amino- with the carboxyl-terminal domain, but also affects the structure and stability of the amino-terminal domain of apoE. The uniqueness of the reduced thermodynamic stability of apoE4-165 compared to the other two alleles suggests that this fragment may have particular structural properties that relate to its function in internalizing Aβ42.

### Analysis of structural and thermodynamic properties of full-length and carboxyl-terminal truncated apoE4 forms

**Figure 7:**
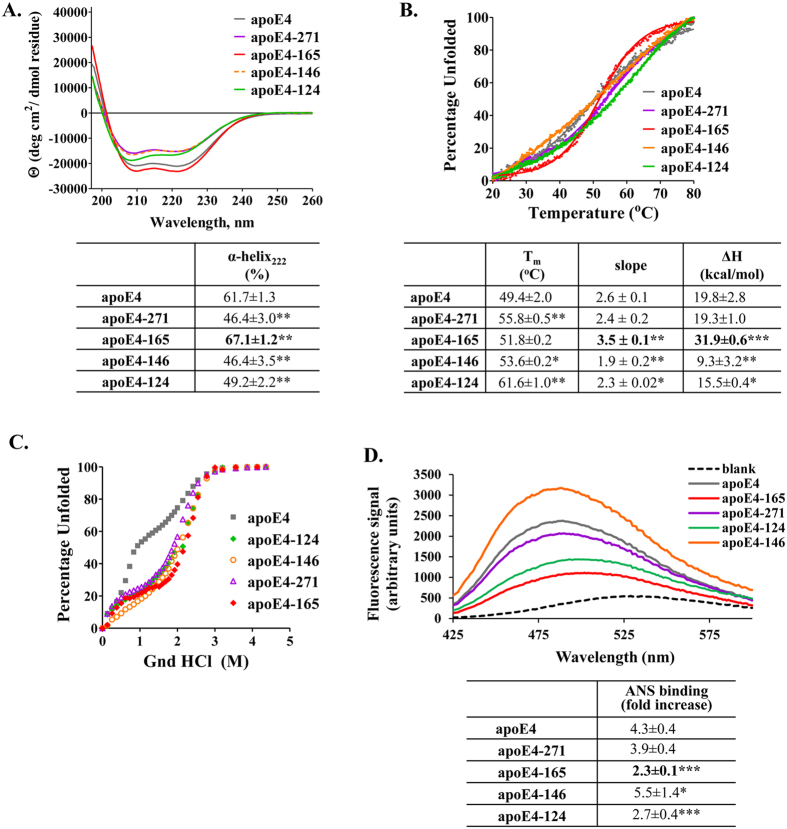
Physicochemical properties of full-length and carboxyl-terminal truncated apoE4 forms. (**A**) Far UV circular dichroism spectra of full-length and truncated apoE4 forms. Spectra are averages of three separate experiments. The % helical content was calculated based on the molar ellipticity at 222 nm as described under “Methods”. **p < 0.005 vs apoE4 (**B**) Thermal denaturation profiles of full-length and truncated apoE4 forms. Y-axis has been normalized to correspond to the percentage of the protein in the unfolded state. Experimental data were fit to a simple two-state Boltzman transition (solid line). Apparent Tm and ΔH values were calculated as described under “Methods”. “Slope” is the calculated slope of the linear component of the thermal denaturation transition around Tm. *p < 0.05, **p < 0.005, ***p < 0.0001 vs apoE4 (**C**) Chemical denaturation profiles of full-length and truncated apoE4 forms. Y-axis has been normalized to correspond to the percentage of the protein in the unfolded state. (**D**) ANS fluorescence spectra in the presence or absence of full-length and truncated apoE4 forms. Spectra are the average of three separate measurements. Fold-increase is the increase in ANS fluorescence in the presence of the protein relative to free ANS in the same buffer. *p < 0.05, ***p < 0.0001 vs apoE4.

The thermodynamic destabilization of apoE4-165 compared to the other two alleles, prompted us to examine whether this fragment also carries different structural properties compared to different length apoE4 fragments not found to have the same bio-activity. Circular dichroism measurements indicate that apoE4-165 is significantly more helical than full-length apoE4 and fragments apoE4-271, apoE4-146 and apoE4-124 (Fig. 7A). Furthermore, apoE4-165 presented a thermal denaturation profile that had a transition mid-point slope that is almost double the slope of other variants (3.5 for apoE4-165 versus 1.9-2.6 for the other variants), indicating a much more cooperative transition and a more compact structure (Fig. 7B). Accordingly, apoE4-165 was the most thermodynamically stable compared to other apoE4 forms with an apparent ΔH at least 12 kcal/mol higher (Fig. 7B). All carboxyl-terminal domain fragments presented a more stabilized chemical denaturation profile compared to full-length apoE4 (Fig. 7C), consistent with a role of the carboxyl-terminal domain in facilitating the unfolding of apoE4 as described before^26^. Finally, hydrophobic surface exposure analysis indicated that apoE4-165 presented the least amount of hydrophobic residues to the solvent (Fig. 7D), again consistent with a more compact structure. Overall, apoE4-165 appears to be unique amongst other length fragments in terms of its structural properties and to present a more compact structure. This is consistent with the folding topology of this fragment that defines a well-structured four helix bundle that sequesters hydrophobic residues from the solvent^24^. These results, in combination with the thermodynamic destabilization of apoE4-165 compared to the ε3 and ε2 alleles, define a unique structural context for this fragment: apoE4-165 seems to have a well-folded compact structure that, however, is more destabilized due to its allelic state presumably facilitating conformational transitions related to its function.

## Discussion

Although it is well established that the ε4 allele of the *APOE* gene increases an individual’s risk for developing late-onset AD, the mechanism by which apoE4 isoform promotes the pathogenesis of the disease has not been elucidated. ApoE fragmentation in the human brain has been shown in numerous studies using mostly immunochemical methods^13^,^14^,^15^,^16^,^17^,^18^,^38^,^39^,^40^, but the exact sequence of the apoE fragments is not known. Several studies showed the presence of carboxyl-terminal truncated forms of apoE4 in brains of AD patients^13^,^14^,^15^,^16^,^17^,^18^ and mice expressing apoE4 in neurons^13^, but not in mice expressing apoE4 in astrocytes^13^. Furthermore, it has been suggested that apoE proteolysis occurs in the secretory and not in the internalization pathway in neurons^13^. ApoE4 proteolysis has been proposed to be an early event in the pathogenesis of AD^13^. In the absence of more detailed experimental information on the exact sequence of apoE4 fragments found in AD patients, we based this study on our previous finding that apoE4-165 can promote the intracellular accumulation of Aβ42 and increase the oxidative stress, two events that have been linked to early pathological processes that lead to AD^20^. This particular fragment has a molecular weight within the range of molecular weights of carboxyl-terminal truncated apoE fragments found in brains of AD patients^14^,^15^,^19^,^40^ and as a result constitutes a useful tool for studying the effects of apoE4 fragments on AD pathogenesis. In the current study we examined whether the functional properties of apoE4-165 are shared with the other isoforms of apoE. Our goal was to gain insight on whether the unique role of apoE4 in the pathogenesis of AD is a direct result of its enhanced proteolytic susceptibility or is rather due to inherent functional properties of this allele. In addition, we aimed to clarify if the fragment length is important or many carboxyl-terminal truncated apoE4 fragments share the same property of internalizing Aβ42. Finally, in an effort to examine whether the unique properties of apoE4-165 fragment are dictated by a distinctive structural conformation, we compared the biophysical and thermodynamic properties of all studied apoE fragments.

Our functional analysis indicates that, at least amongst the fragments evaluated, apoE4-165 is unique in being able to promote the internalization of Aβ42 by human neuroblastoma and primary mouse neuronal cells and to induce intracellular ROS formation. Thermodynamic and structural analysis suggested that apoE4-165 has characteristics that differentiate it from other fragments. Specifically, apoE4-165 folds as a more compact and highly helical structure compared to full-length apoE4 and other carboxyl-terminally truncated fragments, consistent with its length being optimal for the formation of a four-helix bundle that minimizes hydrophobic surface exposure to the solvent. Amongst the three allelic states of apoE-165, however, apoE4-165 is much less thermodynamically stable and has some structural differences. Interestingly, the thermal denaturation profile of apoE4-165 shown in Fig. 6B, suggests that compared to the other two alleles, a significant portion of apoE4-165 (about 10%) may be in an unfolded state at 37 °C. These observations define a unique structural landscape that may underlie the functional properties of apoE4-165: it can fold to a compact four helix bundle that still has significant structural plasticity. It is possible that apoE3-165 and apoE2-165 may not share the functional properties of their apoE4 counterpart because they are too rigid and they cannot undergo structural transitions necessary for function. Indeed, previous analysis has indicated that apoE4-165 may affect the cell membrane micro-fluidity^20^ and therefore, an unfolding transition that would expose the hydrophobic core of the four helix bundle would be expected to facilitate such an interaction. In a recent computational study, it was suggested that apoE4 is distinct from other alleles in being able to form specific misfolded intermediates that have functional consequences^41^. It is therefore conceivable that the unique functional and biophysical properties of apoE4-165 are also related to the formation of allele and size-specific misfolded intermediates that play roles in promoting Aβ42 internalization, although this will have to be investigated further.

In a previous study we found that a similar-length fragment, the apoE4-185, also does not share apoE4-165’s property in internalizing Aβ42. Structurally, apoE4-185 contains the four-helix bundle component of apoE4. Thermodynamic analysis has indicated that apoE4-185 also presents a compact structure, but its thermodynamic stability is higher than that of apoE4-165 and resembles more the apoE3-165 and apoE2-165 (Supplemental Figure 3). As a result, comparison between the similar length fragments apoE4-165 and apoE4-185 in the context of the findings of the current study, strengthens the uniqueness of the structural and thermodynamic properties of apoE4-165 in being a fragment with a compact structure, but with increased conformational plasticity.

The primary function of apoE is to carry lipids and as a result most of apoE exists in a lipidated state. We, however, chose to study the effect of lipid-free apoE4 fragments because previous studies have shown that apoE4-165 (even at the much higher concentration of ~6 μM than this used here) fails to bind and solubilize phospholipid multilamellar vesicles^42^. In addition, lipid-free apoE4-165 does not promote cholesterol efflux via the ATP-binding cassette transporter from SK-N-SH cells at a concentration of 1 μM (unpublished data). Therefore, it is not expected that the carboxyl-terminal truncated apoE-165 fragments, at least at the low apoE concentrations reported for CSF and used here (375 nM), are able to form lipoprotein particles in the brain *in vivo*. It is possible however, that full-length apoE4 and other apoE4 truncated forms scavenge lipids from cells and may be partially lipidated during cell-based assays. Therefore, the exogenously added apoE forms are expected to behave similarly to apoE secreted from brain cells.

The unique functional and structural properties of apoE4-165 suggest that Aβ42 internalization is mediated through a fragment-specific molecular mechanism. One hypothesis would be that apoE4-165 can bind Aβ42 and escort it inside the cell. In a previous study however apoE4-165 failed to bind to Aβ *in vitro*^20^. This is consistent with older studies showing that the carboxyl-terminal region of apoE is necessary for Aβ binding^43^,^44^, and therefore it seems unlikely that apoE4-165 transports Aβ inside the cell by forming a direct molecular complex. In addition, apoE4-165 had no effect on low-density lipoprotein receptor-related protein (LRP) levels and thus Aβ internalization via an LRP-dependent pathway seems also unlikely^20^. ApoE4-165 however, induced a reduction of sphingomyelin levels of SK-N-SH cells, as well as changes in cellular membrane micro-fluidity, possibly affecting the functionality of the cellular membrane^20^. The unique structural properties of apoE4-165 described here are consistent with a mechanism involving the unfolding of the four-helix bundle structure to mediate interactions with the cell membrane facilitating endocytosis of soluble or membrane bound Aβ42. However, one cannot exclude the possibility that the cellular uptake of Aβ proceeds through an unidentified receptor. Additional work will be necessary to clarify the specific mechanism that underlies apoE4-165 mediated Aβ42 internalization by neuronal cells. Furthermore, future studies in mice expressing the apoE4-165 transgene in neurons will be necessary to enhance our understanding of the role of this fragment and apoE4 in AD pathogenesis. Finally, our findings highlight to the necessity of the identification of the exact amino acid sequence of apoE fragments from human brain tissues.

Overall, we show here that apoE4-165 has unique functional and structural properties that may underlie its pathogenic role for AD. The finding that these properties are allele-specific, provide a mechanistic link between two established observations: i) the association of apoE fragmentation with the pathogenesis of AD and ii) the strong association of apoE4 alleles with AD predisposition. Furthermore, our findings suggest that the enhanced proteolytic sensitivity of apoE4 is not an exclusive or even necessary prerequisite for promotion of Aβ42 internalization by neuronal cells. It is possible that the structural characteristics of apoE4 in the context of both easier proteolysis and unique apoE4-165 fragment conformation act synergistically to contribute to AD pathogenesis. Finally, our findings further solidify the mechanistic link between the genetic association of the apoE4 isoform and AD risk and suggest that blocking either the generation or the function of specific apoE4 fragments may be a therapeutic strategy worthwhile exploring.

## Methods

### Materials

Strain BL21-Gold (DE3) of *E. coli* was purchased from Stratagene (Cedar Creek, TX, USA). The Complete mini EDTA-free protease inhibitor cocktail was from Roche (Mannheim, Germany). Nickel-nitrilotriacetic acid (Ni-NTA) resin was purchased from Thermo Scientific (Rockford, IL, USA). Lyophilized Aβ(1–42), HFIP treated, was from JPT Peptide Technologies GmbH (Berlin, Germany). Cell culture media and other reagents were purchased from Sigma Aldrich (St. Louis, MO, USA), Biochrom AG (Berlin, Germany), Lonza (Verviers, Belgium), Bio-Rad (Hercules, CA, USA), Fisher Scientific (Schwerte, Germany), GE Healthcare (Uppsala, Sweden) or other standard commercial sources.

### Site direct mutagenesis

The pET32-E4/3C and pET32-E3/3C vectors containing a thioredoxin (Trx) tag, a 6x His-tag and a 3C-protease site at the fusion junction with the human cDNA for full-length apoE4 or apoE3 have been described previously^32^,^33^. To generate the vector for expression of the apoE2 isoform, we used site-directed mutagenesis to introduce the R158C mutation in the apoE3 gene, generating the pET32-E2/3C plasmid. The mutagenesis reaction was performed using the QuickChange II XL site direct mutagenesis kit (Agilent; Santa Clara, CA) according to the manufacturer’s instructions. The primers used for the mutagenesis are: apoER158C-for (5′-C GAT GAC CTG CAG AAG **TGC** CTG GCA GTG TAC CAG G -3′) and apoER158C-rev (5′-C CTG GTA CAC TGC CAG **GCA** CTT CTG CAG GTC ATC G-3′).

Using the same approach, we introduced a stop codon after the coding triplet for amino acid 165 in the pET32-E2/3C, pET32-E3/3C and pET32-E4/3C vectors with the mutagenesis primers delta166-for (5′-GCA GTG TAC CAG GCC GGG **TAA** CGC GAG GGC GCC GAG CG-3′) and delta166-rev (5′-CG CTC GGC GCC CTC GCG **TTA** CCC GGC CTG GTA CAC TGC-3′). In addition, we introduced a stop codon after the coding triplet for amino acids 271, 146 and 124 in the pET32-E4/3C vector. The sequences of the primers used for the mutagenesis are: delta272-for (5′-C CTG GTG GAA GAC **TAG** CAG CGC CAG TGG GCC G-3′); delta272-rev (5′-C GGC CCA CTG GCG CTG **CTA** GTC TTC CAC CAG G-3′); delta147-for (5′-CAC CTG CGC AAG CTG CGT AAG **TAA** CTC CTC CGC GAT GCC-3′); delta147-rev (5′-GGC ATC GCG GAG GAG **TTA** CTT ACG CAG CTT GCG CAG GTG-3′); delta125-for (5′-GC GAG GTG CAG GCC **TAA** CTC GGC CAG AGC ACC GAG-3′); delta125-rev (5′CTC GGT GCT CTG GCC GAG **TTA** GGC CTG CAC CTC GC-3′). Successful mutagenesis was confirmed by DNA sequencing.

### Expression and purification of full-length and carboxyl-terminal truncated apoE forms

The expression and purification of apoE4 and apoE3 was carried out as described previously^32^,^33^. ApoE2 was expressed and purified following the same protocol. ApoE2-165, apoE3-165, apoE4-165, apoE4-271, apoE4-146 and apoE4-124 truncated forms were also expressed and purified following the same protocol with some modifications. Briefly, BL21-Gold(DE3) cells were transformed with the expression vectors, described above, carrying the sequence of the gene of carboxyl-terminal truncated mutants (apoE2-165, apoE3-165, apoE4-165, apoE4-271, apoE4-146 and apoE4-124) and cultured in LB medium containing 100 μg/ml ampicillin at 37 °C. Protein expression was induced with IPTG (final concentration 0.5 mM) for 2 h. Cells were lysed in 20 mM Tris-HCl buffer, pH 8.0 containing 0.5 M NaCl, complete mini EDTA-free protease inhibitors cocktail and 0.1 mg/ml lysozyme (40 ml per 1 L of original culture) by using a French Press (SLM-AMINCO, USA) and the lysate was centrifuged to remove cellular debris. The Trx-fused apoE carboxyl-terminal truncated forms in the supernatant were purified by Ni-NTA chromatography as follows: the supernatant was adjusted to contain 20 mM Tris-HCl, pH 8.0, 0.5 NaCl, and 5 mM imidazole and incubated with 4 ml (per 1L of original culture) Ni-NTA resin, under gentle stirring, at 4 °C overnight. The following day, the Ni-NTA suspension was loaded onto an empty chromatography column and washed with 10 mM Tris-HCl, pH 8.0, 0.5 M NaCl, 5 or 10 mM imidazole. The Trx-fused apoE2-165, apoE3-165, apoE4-165 and apoE4-271 were eluted with 20 mM Tris-HCl, pH 8.0, 0.5 M NaCl containing 50–300 mM imidazole. The Trx-fused apoE4-125 was eluted with 20 mM Tris-HCl, pH 8.0, 0.5 M NaCl containing 20–300 mM imidazole and the Trx-fused apoE4-147 was eluted with 20 mM Tris-HCl, pH 8.0, 0.5 M NaCl containing 50–150 mM imidazole. The collected fractions were dialyzed extensively against 20 mM Tris-HCl, pH 8.0, 0.5 M NaCl, at 4 °C. Following dialysis, His-tagged 3C protease (prepared as described previously^32^ using the vector pET-24/His-3C that was kindly provided by Dr. Arie Geerlof (EMBL, Heidelberg, Germany)) was added at a ratio of 1/70 (3C-protease/Trx-apoE, w/w) and cleavage of Trx from apoE carboxyl-terminal truncated forms was allowed to proceed for 18 h at 4 °C in 20 mM Tris-HCl buffer, pH 8.0 containing 0.5 M NaCl and 1 mM DTT. The apoE carboxyl-terminal truncated forms were separated by the cleaved tag by a second Ni-NTA resin affinity chromatography step. To facilitate the dissociation of noncovalent complexes among any uncut apoE, cut apoE and cut Trx the cleavage reaction protein solution was adjusted by adding 6 M urea (final concentration) and the solution was incubated with 1 ml of Ni-NTA resin (per 4 mg of protein) for 1 h at 4 °C. The suspension was loaded onto an empty chromatography column and the cut apoE carboxyl-terminal truncated forms were found in the column flow-through. Each apoE solution was extensively dialyzed against 5 mM NH_4_HCO_3_, lyophilized, and stored at −80 °C. Before analyses, lyophilized stocks of proteins were dissolved in 6 M guanidine hydrochloride (GndHCl) in 50 mM sodium phosphate buffer pH 7.4 containing 1 mM DTT and refolded by extensive dialysis against 50 mM sodium phosphate buffer pH 7.4, 1 mM DTT, as described previously^32^,^33^. Protein concentrations were determined either by absorbance measurements at 280 nm or by the Lowry procedure (DC Protein Assay Kit, Bio-Rad, Hercules, CA, USA). The refolded proteins were at least 95–98% pure, as estimated by SDS-PAGE and staining with Coomassie Brilliant Blue. ApoE-165 forms were also analyzed by native 15% PAGE followed by Coomassie Brilliant Blue staining.

### Circular dichroism (CD) measurements

Far-UV CD spectra were recorded as described previously^29^ using a Jasco 715 (USA) spectropolarimeter at 20 °C in a 1 mm path-length quartz cuvette. The concentration of the protein samples was 0.1 mg/ml in DPBS (pH 7.4). Spectra were recorded from 195 to 260 nm by using the following measurement parameters: bandwidth 1 nm, response 8 s, step size 0.2 nm and scan speed 50 nm/min. During the measurements a Jasco PTC-348 WI Peltier temperature controller was connected to the instrument for thermostating the cuvette chamber. Each spectrum was the average of five accumulations. All spectra were obtained by subtracting the buffer baseline. Helical content was calculated using the molecular ellipticity at 222 nm using the equation^45^:


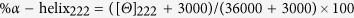


For thermal denaturation measurements, the change in molar ellipticity at 222 nm was monitored while varying the temperature in the range 20–80 °C at a rate of 1 °C/min. The thermal denaturation curve was fitted to a Boltzman simple sigmoidal model curve using the Graphpad Prism™ software (GraphPad Software Inc, La Jolla, CA, USA). The apparent melting temperature T_m_ was determined by the sigmoidal fit as midpoint of the thermal transition. The relative enthalpy change was calculated as described previously^46^. The slope of the linear component of the thermal denaturation transition, around the melting temperature T_m_, is also reported.

### Chemical denaturation experiments

To record the chemical denaturation profile of apoE samples we measured the changes in intrinsic tryptophan fluorescence (excitation 295, emission 340 nm) of the proteins in solution upon addition of increasing amounts of 8.0 M guanidine hydrochloride (GndHCl). Briefly, 0.05 mg/ml of freshly refolded protein were inserted in a 4 mL quartz fluorometer cuvette, small amounts of an 8.0 M GndHCl was gradually added to the solution and the contents were mixed by repeated pipetting for 5 s. Then, the mixture was incubated for 2 min in the dark and then the fluorescence signal of the sample was measured in a Quantamaster 4 fluorescence spectrometer (Photon Technology International, New Jersey, USA). For the apoE-165 truncated forms, the chemical denaturation curve obtained between 0.5–4.2 M GndHCl was fitted to a Boltzman simple sigmoidal model, using the Graphad Prism™ software, in order to facilitate the calculation of the midpoint of the main transition, D_1/2_.

### ANS fluorescence measurement

1-anilinonaphthalene-8-sulfonic acid (1,8 ANS, Sigma-Aldrich) was dissolved in dimethylsulfoxide to a final concentration of 50 mM (ANS stock solution) and stored at −20 °C. Freshly refolded apoE samples at 0.0625 mg/mL were placed into the wells of a 96-well black microplate, and the fluorescence signal was measured by an Infinite M200 microplate reader (Tecan group Ltd., Mannedorf, Switzerland). The excitation wavelength was set at 395 nm and the emission range from 425 to 600 nm. One microliter of ANS stock solution was added to each sample and mixed so that the final ANS concentration was 310 μM and the fluorescence signal was recorded. A control ANS spectrum in the absence of protein was also recorded to allow the calculation of ANS fluorescence enhancement in the presence of apoE forms.

### Tryprophan fluorescence quenching measurement

Tryptophan fluorescence quenching experiments were performed on freshly refolded apoE-165 using pyrene (Sigma-Aldrich) as an external quencher^47^. 100 μl of apoE-165 samples at 0.1 mg/mL were placed into the wells of a 96-well black microplate and mixed thoroughly with 1 μl of pyrene stock solutions to achieve a final concentration of 6.25–50 μM. Following 3 min incubation in the dark, the fluorescence signal was measured by an Infinite M200 microplate reader (Tecan group Ltd.,). The excitation wavelength was set at 286 nm and the emission wavelength was set at 340 nm. The control fluorescence signal of each protein in the absence of pyrene was also recorded to allow the calculation of fluorescence quenching in the presence of pyrene. Additionally, the fluorescence intensity of pyrene at 340 nm in the absence of protein was recorded and subtracted from the signal of each sample.

### Dynamic Light Scattering (DLS) Analysis

DLS analysis of freshly refolded apoE-165 samples at 0.1 mg/mL was performed using a Zetasizer nano series instrument (Malvern Instruments Ltd, UK) at 20 °C.

### Cell Cultures

Human neuroblastoma SK-N-SH cells (ATCC, Rockville, MD, USA) were cultured in minimum essential medium (MEM) Earle’s supplemented with 2 mM L-glutamine, 0.1 mM nonessential amino acids, 1 mM sodium pyruvate, 1.5 g/L sodium bicarbonate, 10% FBS (MEM-Earle’s complete) and antibiotics.

Primary cultures of mouse cortical neurons were prepared from postnatal day 0 male pups of C57BL/6 mice as described^36^,^48^. Briefly, the cortices were dissected, neurons were dissociated by Trypsin/DNase digestion and the cells were plated at a density of 6.6 × 10^4^/cm^2^ on glass coverslips coated with poly-D-lysine (12 mm, no. 1 thickness, VitroCam, London, UK) and cultured in 0.5 ml Neurobasal medium supplemented with 2% B-27 serum-free supplement, 0.5 mM Glutamax, 100 U/ml penicillin G and 100 mg/ml streptomycin sulfate.

### Confocal microscopy

SK-N-SH cells or primary mouse cortical neurons seeded on coverslips were incubated with 25 ng/ml Aβ42 in the presence or absence of 0.375 μM lipid-free full-length or truncated apoE forms in serum-free (or B-27 supplement-free) cell medium for 24 h at 37 °C or after removal of apoE forms and Aβ42 from the cell medium for 24 more hours at 37 °C. At the end of incubation, cells were washed with PBS, fixed with 4% paraformaldehyde at room temperature for 15 minutes, washed again with PBS and incubated in blocking and permeabilization buffer (PBS/5% FBS/0.05% Tween-20) for 30 min at 25 °C. Aβ42 was stained by using primary mouse anti-Aβ monoclonal antibody 6E10 (1:200, Chemicon, Temecula, CA, USA) and secondary Alexa Fluor 488-conjugated goat anti-mouse IgG antibody (1:1000, Invitrogen, Carlsbad, CA, USA). For F-actin staining rhodamine phalloidin (1:200, Cytoskelton Inc, Denver, CO, USA) was used. Coverslips were mounted onto slides and viewed with a Bio-Rad MRC 1024ES laser scanning confocal microscope (Bio-Rad, Herculles, CA, USA) mounted on a Nikon Eclipse E600 upright microscope equipped with Krypton-Argon laser, a motor step of 0.5 μm and the Lasersharp Acquisition software (Windows NT Operating System). Images were acquired, maintaining fixed laser intensities and camera settings. Images were analyzed using the FIJI image analysis software^49^, after appropriate thresholding, to quantify the fluorescence intensity. Relative fluorescent intensity was measured for 3–8 images acquired in each experimental condition.

### Measurement of ROS generation

Intracellular ROS generation was measured by following a previously described method^50^ modified for fluorescent microscopy. SK-N-SH cells were plated on 24-well plates at a density of 1 × 10^5^ cells/well in MEM-Earle’s complete cell medium and cultured until 90% confluence. Cells were then incubated with 25 ng/ml Aβ42 in the presence or absence of 0.375 μM lipid-free full-length or truncated apoE forms in serum-free cell medium for 24 h at 37 °C. At the end of the incubation period the cells were washed with DMEM and incubated in the dark for 45 min at 37 °C in DMEM containing 25 μΜ 2′,7′-dichlorofluorescin diacetate (DCFH-DA, Molecular probes/Invitrogen). Subsequently, the cells were washed with preheated at 37 °C PBS and the production of ROS was detected by recording the fluorescence of 2′,7′-dichlorofluorescein (DCF) with the Axiovert 25 (Zeiss, Gottingen, Germany) inverted microscope equipped for fluorescence microscopy (excitation 450-490 nm, emission 520 nm). DCF fluorescent intensity was measured for at least 40 cells from the fluorescent images of each sample using the ImageJ image analysis software (NIH, Bethesda, MD, USA)^51^ and the relative fluorescence intensity was taken as average of the values of at least 5 images for each experiment.

## Additional Information

**How to cite this article**: Dafnis, I. *et al*. The ability of apolipoprotein E fragments to promote intraneuronal accumulation of amyloid beta peptide 42 is both isoform and size-specific. *Sci. Rep.* **6**, 30654; doi: 10.1038/srep30654 (2016).

## Supplementary Material

Supplementary Information

## References

[b1] Jarrett J. T., Berger E. P. & Lansbury P. T. Jr. The carboxy terminus of the beta amyloid protein is critical for the seeding of amyloid formation: implications for the pathogenesis of Alzheimer’s disease. 32, 4693–4697 (1993). 10.1021/bi00069a001 8490014

[b2] Selkoe D. J. Alzheimer’s disease. Cold Spring. Harb. Perspect. Biol. 3, a004457 (2011). 21576255 10.1101/cshperspect.a004457 PMC3119915

[b3] Musiek E. S. & Holtzman D. M. Three dimensions of the amyloid hypothesis: time, space and ‘wingmen’. 18, 800–806 (2015). 10.1038/nn.4018 PMC4445458 26007213

[b4] LaFerla F. M., Green K. N. & Oddo S. Intracellular amyloid-beta in Alzheimer’s disease. 8, 499–509 (2007). 10.1038/nrn2168 17551515

[b5] Baker-Nigh A. . Neuronal amyloid-beta accumulation within cholinergic basal forebrain in ageing and Alzheimer’s disease. 138, 1722–1737 (2015). 10.1093/brain/awv024 PMC4542619 25732182

[b6] Bayer T. A. & Wirths O. Review on the APP/PS1KI mouse model: intraneuronal Abeta accumulation triggers axonopathy, neuron loss and working memory impairment. Genes Brain Behav. 7 Suppl 1, 6–11 (2008). 18184366 10.1111/j.1601-183X.2007.00372.x

[b7] Scala F. . Intraneuronal Abeta accumulation induces hippocampal neuron hyperexcitability through A-type K(+) current inhibition mediated by activation of caspases and GSK-3. Neurobiol. Aging. 36, 886–900 (2015). 25541422 10.1016/j.neurobiolaging.2014.10.034 PMC4801354

[b8] Harold D. . Genome-wide association study identifies variants at CLU and PICALM associated with Alzheimer’s disease. 41, 1088–1093 (2009). 10.1038/ng.440 PMC2845877 19734902

[b9] Lambert J. C. . Genome-wide association study identifies variants at CLU and CR1 associated with Alzheimer’s disease. 41, 1094–1099 (2009). 10.1038/ng.439 19734903

[b10] Mahley R. W., Weisgraber K. H. & Huang Y. Apolipoprotein E4: a causative factor and therapeutic target in neuropathology, including Alzheimer’s disease. 103, 5644–5651 (2006). 10.1073/pnas.0600549103 PMC1414631 16567625

[b11] Zannis V. I. . “Lipoproteins and Atherogenesis,“in *Molecular Mechanisms of Atherosclerosis*, edited by Loscalzo J. (Taylor & Francis, Abington, UK), pp.111–174 (2004).

[b12] Zannis V. I., Cole F. S., Jackson C. L., Kurnit D. M. & Karathanasis S. K. Distribution of apolipoprotein A-I, C-II, C-III, and E mRNA in fetal human tissues. Time-dependent induction of apolipoprotein E mRNA by cultures of human monocyte-macrophages. 24, 4450–4455 (1985). 10.1021/bi00337a028 3931677

[b13] Brecht W. J. . Neuron-specific apolipoprotein e4 proteolysis is associated with increased tau phosphorylation in brains of transgenic mice. 24, 2527–2534 (2004). 10.1523/JNEUROSCI.4315-03.2004 PMC6729489 15014128

[b14] Harris F. M. . Carboxyl-terminal-truncated apolipoprotein E4 causes Alzheimer’s disease-like neurodegeneration and behavioral deficits in transgenic mice. 100, 10966–10971 (2003). 10.1073/pnas.1434398100 PMC196910 12939405

[b15] Huang Y. . Apolipoprotein E fragments present in Alzheimer’s disease brains induce neurofibrillary tangle-like intracellular inclusions in neurons. 98, 8838–8843 (2001). 10.1073/pnas.151254698 PMC37522 11447277

[b16] Jones P. B. . Apolipoprotein E: isoform specific differences in tertiary structure and interaction with amyloid-beta in human Alzheimer brain. PLoS. ONE. 6, e14586 (2011). 21297948 10.1371/journal.pone.0014586 PMC3031506

[b17] Rohn T. T., Catlin L. W., Coonse K. G. & Habig J. W. Identification of an amino-terminal fragment of apolipoprotein E4 that localizes to neurofibrillary tangles of the Alzheimer’s disease brain. Brain Res. 1475, 106–115 (2012). 22902767 10.1016/j.brainres.2012.08.003

[b18] Wang M. & Turko I. V. Mass spectrometry quantification revealed accumulation of C-terminal fragment of apolipoprotein E in the Alzheimer’s frontal cortex. 8, e61498 (2013). 10.1371/journal.pone.0061498 PMC3623866 23593485

[b19] Cho H. S., Hyman B. T., Greenberg S. M. & Rebeck G. W. Quantitation of apoE domains in Alzheimer disease brain suggests a role for apoE in Abeta aggregation. 60, 342–349 (2001). 10.1093/jnen/60.4.342 11305869

[b20] Dafnis I. . An apolipoprotein E4 fragment can promote intracellular accumulation of amyloid peptide beta 42. 115, 873–884 (2010). 10.1111/j.1471-4159.2010.06756.x PMC2910182 20412390

[b21] Morrow J. A. . Apolipoprotein E4 forms a molten globule. A potential basis for its association with disease. 277, 50380–50385 (2002). 10.1074/jbc.M204898200 12393895

[b22] Aggerbeck L. P., Wetterau J. R., Weisgraber K. H., Wu C. S. & Lindgren F. T. Human apolipoprotein E3 in aqueous solution. II. Properties of the amino- and carboxyl-terminal domains. 263, 6249–6258 (1988). 3360782

[b23] Wetterau J. R., Aggerbeck L. P., Rall S. C. Jr. & Weisgraber K. H. Human apolipoprotein E3 in aqueous solution. I. Evidence for two structural domains. 263, 6240–6248 (1988). 3360781

[b24] Dong L. M. . Human apolipoprotein E. Role of arginine 61 in mediating the lipoprotein preferences of the E3 and E4 isoforms. 269, 22358–22365 (1994). 8071364

[b25] Wilson C., Wardell M. R., Weisgraber K. H., Mahley R. W. & Agard D. A. Three-dimensional structure of the LDL receptor-binding domain of human apolipoprotein E. 252, 1817–1822 (1991). 10.1126/science.2063194 2063194

[b26] Hatters D. M., Peters-Libeu C. A. & Weisgraber K. H. Apolipoprotein E structure: insights into function. Trends Biochem. Sci. 31, 445–454 (2006). 16820298 10.1016/j.tibs.2006.06.008

[b27] Mahley R. W., Weisgraber K. H. & Huang Y. Apolipoprotein E: structure determines function, from atherosclerosis to Alzheimer’s disease to AIDS. 50 Suppl, S183–S188 (2009). 10.1194/jlr.R800069-JLR200 PMC2674716 19106071

[b28] Argyri L. . Molecular basis for increased risk for late-onset Alzheimer disease due to the naturally occurring L28P mutation in apolipoprotein E4. 289, 12931–12945 (2014). 10.1074/jbc.M113.538124 PMC4007480 24644280

[b29] Chroni A. . Biophysical analysis of progressive C-terminal truncations of human apolipoprotein E4: insights into secondary structure and unfolding properties. 47, 9071–9080 (2008). 10.1021/bi800469r PMC2692411 18690708

[b30] Morrow J. A. . Differences in stability among the human apolipoprotein E isoforms determined by the amino-terminal domain. 39, 11657–11666 (2000). 10.1021/bi000099m 10995233

[b31] Bien-Ly N. . C-terminal-truncated apolipoprotein (apo) E4 inefficiently clears amyloid-beta (Abeta) and acts in concert with Abeta to elicit neuronal and behavioral deficits in mice. Proc. Natl. Acad. Sci. USA 108, 4236–4241 (2011). 21368138 10.1073/pnas.1018381108 PMC3053957

[b32] Argyri L., Skamnaki V., Stratikos E. & Chroni A. A simple approach for human recombinant apolipoprotein E4 expression and purification. 79, 251–257 (2011). 10.1016/j.pep.2011.06.011 21712092

[b33] Georgiadou D. . Thermodynamic and structural destabilization of apoE3 by hereditary mutations associated with the development of lipoprotein glomerulopathy. 54, 164–176 (2013). 10.1194/jlr.M030965 PMC3520522 23110818

[b34] Kay A. D. . Cerebrospinal fluid apolipoprotein E concentration decreases after traumatic brain injury. J. Neurotrauma 20, 243–250 (2003). 12820678 10.1089/089771503321532824

[b35] Wang L. . Cerebrospinal fluid apolipoprotein E concentration decreases after seizure. Seizure. 19, 79–83 (2010). 20093050 10.1016/j.seizure.2009.12.001

[b36] Li J. . Differential regulation of amyloid-beta endocytic trafficking and lysosomal degradation by apolipoprotein E isoforms. 287, 44593–44601 (2012). 10.1074/jbc.M112.420224 PMC3531774 23132858

[b37] Georgiadou D., Chroni A., Vezeridis A., Zannis V. I. & Stratikos E. Biophysical Analysis of Apolipoprotein E3 Variants Linked with Development of Type III Hyperlipoproteinemia. PLoS. ONE. 6, e27037 (2011). 22069485 10.1371/journal.pone.0027037 PMC3206067

[b38] Conejero-Goldberg C. . APOE2 enhances neuroprotection against Alzheimer’s disease through multiple molecular mechanisms. Mol. Psychiatry. 19, 1243–1250 (2014). 24492349 10.1038/mp.2013.194

[b39] Elliott D. A. . Isoform-specific proteolysis of apolipoprotein-E in the brain. Neurobiol. Aging. 32, 257–271 (2011). 19278755 10.1016/j.neurobiolaging.2009.02.006

[b40] Marques M. A., Tolar M., Harmony J. A. & Crutcher K. A. A thrombin cleavage fragment of apolipoprotein E exhibits isoform-specific neurotoxicity. Neuroreport 7, 2529–2532 (1996). 8981417 10.1097/00001756-199611040-00025

[b41] Williams I. B., Convertino M., Das J. & Dokholyan N. V. ApoE4-specific Misfolded Intermediate Identified by Molecular Dynamics Simulations. PLoS Comput. Biol. 11, e1004359 (2015). 26506597 10.1371/journal.pcbi.1004359 PMC4623519

[b42] Li X., Kypreos K., Zanni E. E. & Zannis V. Domains of apoE required for binding to apoE receptor 2 and to phospholipids: Implications for the functions of apoE in the brain. 42, 10406–10417 (2003). 10.1021/bi027093c 12950167

[b43] Aleshkov S. B., Li X., Lavrentiadou S. N. & Zannis V. I. Contribution of cysteine 158, the glycosylation site threonine 194, the amino- and carboxy-terminal domains of apolipoprotein E in the binding to amyloid peptide beta (1–40). 38, 8918–8925 (1999). 10.1021/bi982002q 10413465

[b44] Pillot T. . Beta-amyloid peptide interacts specifically with the carboxy-terminal domain of human apolipoprotein E: relevance to Alzheimer’s disease. 72, 230–237 (1999). 10.1046/j.1471-4159.1999.0720230.x 9886074

[b45] Morrisett J. D., David J. S., Pownall H. J. & Gotto A. M. Jr. Interaction of an apolipoprotein (apoLP-alanine) with phosphatidylcholine. 12, 1290–1299 (1973). 10.1021/bi00731a008 4348832

[b46] Gorshkova I. N., Liadaki K., Gursky O., Atkinson D. & Zannis V. I. Probing the lipid-free structure and stability of apolipoprotein A-I by mutation. 39, 15910–15919 (2000). 10.1021/bi0014406 11123918

[b47] Maity A., Mukherjee P., Das T., Ghosh P. & Purkayastha P. Forster resonance energy transfer between pyrene and bovine serum albumin: effect of the hydrophobic pockets of cyclodextrins. Spectrochim. Acta A. Mol. Biomol. Spectrosc. 92, 382–387 (2012). 22446788 10.1016/j.saa.2012.02.088

[b48] Lesuisse C. & Martin L. J. Long-term culture of mouse cortical neurons as a model for neuronal development, aging, and death. J. Neurobiol. 51, 9–23 (2002). 11920724 10.1002/neu.10037

[b49] Schindelin J. . Fiji: an open-source platform for biological-image analysis. Nat. Methods 9, 676–682 (2012). 22743772 10.1038/nmeth.2019 PMC3855844

[b50] Bae Y. S. . Epidermal growth factor (EGF)-induced generation of hydrogen peroxide. Role in EGF receptor-mediated tyrosine phosphorylation. 272, 217–221 (1997). 8995250

[b51] Abramoff M. D., Magelhaes P. J. & Ram S. J. Image Processing with ImageJ. 11, 36–42 (2004).

