# Supplementary material for: The ability of apolipoprotein E fragments to promote intraneuronal accumulation of amyloid beta peptide 42 is both isoform and size-specific
Source: Sci Rep. 2016 Aug 1;6:30654. doi: 10.1038/srep30654 (PMC4967930; doi:10.1038/srep30654)
Supplement: Supplementary Information [file srep30654-s1.pdf]

The ability of apolipoprotein E fragments to promote intraneuronal accumulation of amyloid beta peptide 42 is both isoform and size-specific

Ioannis Dafnis, Letta Argyri, Marina Sagnou, Athina Tzinia, Effie C. Tsilibary, Efstratios Stratikos and Angeliki Chroni

SUPPLEMENTAL FIGURES

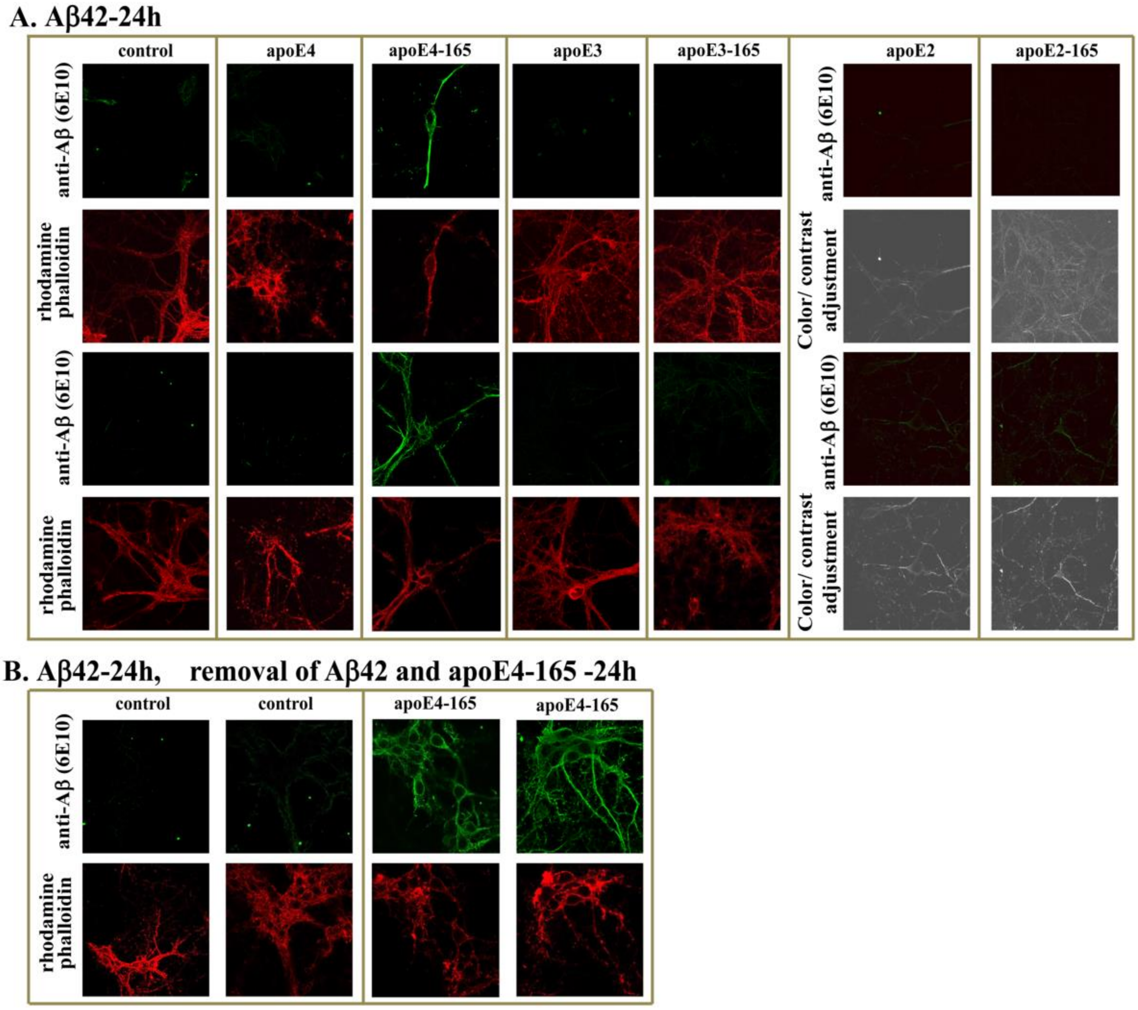

Supplemental Figure 1. Fluorescence confocal laser scanning microscopy of primary mouse cortical neurons incubated in the presence of Aβ42 and full-length apoE or truncated apoE-165 forms.

A. Primary mouse cortical neurons were incubated with 25 ng/ml Aβ42 in the absence (control) or presence of 375 nM lipid-free full-length apoE or apoE-165 forms for 24 h, as indicated in each panel.

B. Primary mouse cortical neurons were also incubated with 25 ng/ml Aβ42 in the absence (control) or presence of 375 nM lipid-free apoE4-165 for 24 h and then washed and incubated further in fresh medium without Aβ42 or apoE4-165 for 24 more hours, as indicated. Aβ immunostaining of cells was detected with the antibody 6E10 followed by a FITC-conjugated secondary antibody (green). The F-actin staining of cells using rhodamine phalloidin (red) or greyscale images of increased brightness/contrast are shown to facilitate the visualisation of cells outline, especially in the images with very low Aβ immunostaining.

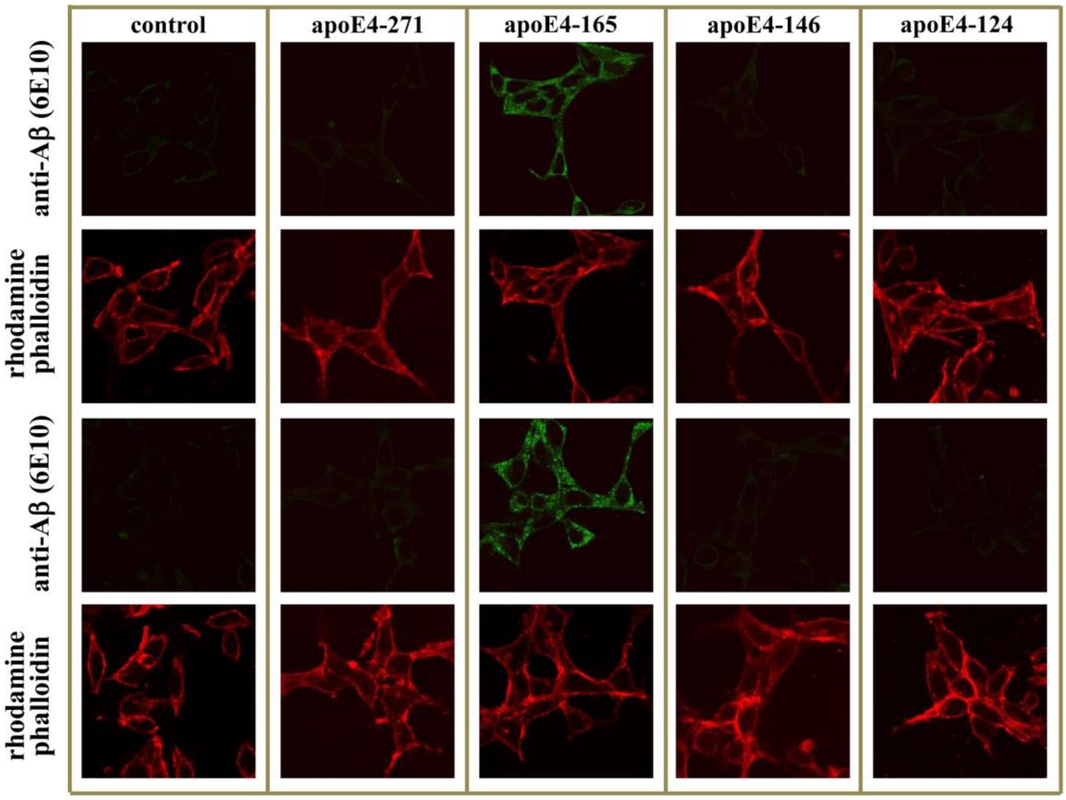

Supplemental Figure 2. Fluorescence confocal laser scanning microscopy of SK-N-SH cells incubated in the presence of Aβ42 and carboxy-terminal truncated apoE4 forms. SK-N-SH cells were incubated with 25 ng/ml Aβ42 in the absence (control) or presence of 375 nM lipid-free carboxyl-terminal truncated apoE4 forms for 24 h, as indicated in each panel. Aβ immunostaining of cells was detected with the antibody 6E10 followed by an FITC-conjugated secondary antibody (green). The F-actin staining of cells using rhodamine phalloidin (red) is shown to facilitate the visualisation of cells outline, especially in the images with very low Aβ immunostaining.

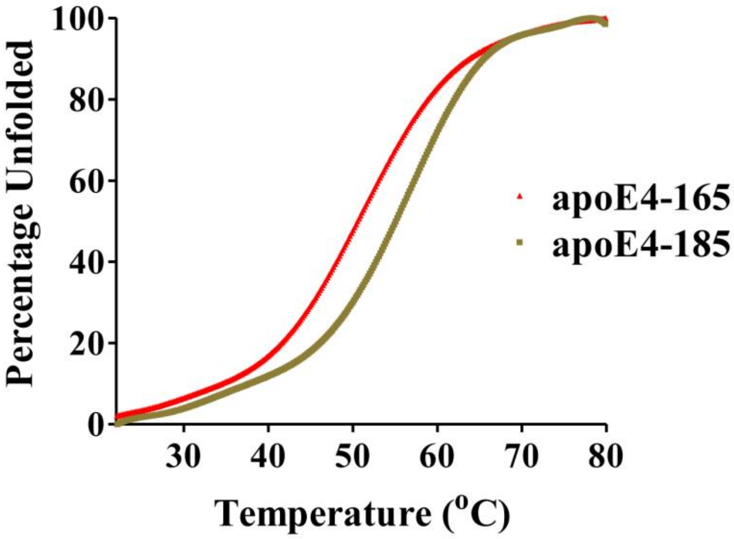

	Thermal denaturation			ANS binding
	**Tm (^{o}C) [1]**	**slope**	**ΔH (Kcal/mol)**	**(fold increase) [1]**
**apoE4-185**	56.9	4.0	36.7	1.5
**apoE4-165**	**51.1**	**3.7**	**33.6**	**2.3**

[1] The values have been published in: Chroni, A., Pyrpassopoulos, S., Thanassoulas, A., Nounesis, G., Zannis, V. I., and Stratikos, E. (2008) Biophysical analysis of progressive C-terminal truncations of human apolipoprotein E4: insights into secondary structure and unfolding properties, *Biochemistry 47*, 9071-9080.

Supplemental Figure 3. Physicochemical properties of the carboxyl-terminal truncated apoE4 forms apoE4-165 and apoE4-185. The graph shows the thermal denaturation profiles of apoE4-165 and apoE4-185. Y-axis has been normalized to correspond to the percentage of the protein in the unfolded state. Experimental data were fit to a simple two-state Boltzman transition. Apparent Tm values have been published previously (Chroni et al, Biochemistry 2008). ΔH values were calculated as described under "Materials and Methods". "Slope" is the calculated slope of the linear component of the thermal denaturation transition around Tm. Fold-increase values that show the increase in ANS fluorescence in the presence of the protein relative to free ANS in the same buffer have been published previously (Chroni et al, Biochemistry 2008).
